# Study of early warning for desaturation provided by Oxygen Reserve Index in obese patients

**DOI:** 10.1007/s10877-020-00531-w

**Published:** 2020-05-18

**Authors:** Ekaterina Tsymbal, Sebastian Ayala, Amrik Singh, Richard L. Applegate, Neal W. Fleming

**Affiliations:** Department of Anesthesiology and Pain Medicine, University of California Davis, 4150 V Street, PSSB Suite 1200, Sacramento, CA 95817 USA

**Keywords:** Arterial desaturation, Endotracheal intubation, Oxygen reserve index, Preoxygenation, Pulse oximetry

## Abstract

Acute hemoglobin desaturation can reflect rapidly decreasing PaO_2_. Pulse oximetry saturation (SpO_2_) facilitates hypoxia detection but may not significantly decrease until PaO_2_ < 80 mmHg. The Oxygen Reserve Index (ORI) is a unitless index that correlates with moderately hyperoxic PaO_2_. This study evaluated whether ORI provides added arterial desaturation warning in obese patients. This IRB approved, prospective, observational study obtained written informed consent from Obese (body mass index (BMI) kg m^−2^; 30 < BMI < 40) and Normal BMI (19 < BMI < 25) adult patients scheduled for elective surgery requiring general endotracheal anesthesia. Standard monitors and an ORI sensor were placed. Patient’s lungs were pre-oxygenated with 100% FiO_2_. After ORI plateaued, general anesthesia was induced, and endotracheal intubation accomplished using a videolaryngoscope. Patients remained apneic until SpO_2_reached 94%. ORI and SpO_2_ were recorded continuously. Added warning time was defined as the difference between the time to SpO_2_ 94% from ORI alarm start or from SpO_2_ 97%. Data are reported as median; 95% confidence interval. Complete data were collected in 36 Obese and 36 Normal BMI patients. ORI warning time was always longer than SpO_2_ warning time. Added warning time provided by ORI was 46.5 (36.0–59.0) seconds in Obese and 87.0 (77.0–109.0) seconds in Normal BMI patients, and was shorter in Obese than Normal BMI patients difference 54.0 (38.0–74.0) seconds (p < 0.0001). ORI provided what was felt to be clinically significant added warning time of arterial desaturation compared to SpO_2_. This added time might allow earlier calls for help, assistance from other providers, or modifications of airway management.

*Trial registration* ClinicalTrials.gov NCT03021551.

## Introduction

Prolonged hypoxemia can produce tissue injury and serious perioperative complications including dysrhythmias, brain injury or death [1]. Prior to widespread utilization of pulse oximetry, hypoxemia was the leading cause of anesthesia-associated perioperative mortality worldwide [2]. Assessment of a patients’ oxygenation to detect desaturations early facilitates interventions that prevent complications of prolonged hypoxemia [3]. Arterial blood gas (ABG) analysis provides a definitive measurement of oxygenation status [4], but is limited by its intermittent and invasive nature.

Pulse oximetry is noninvasive, and provides efficient cost-effective monitoring, so it is often used in lieu of ABG analysis [4]. Pulse oximetry oxygen saturation (SpO_2_) is determined by measuring the absorption of two emitted light wavelengths (660 nm and 940 nm) and calculating the proportion of oxyhemoglobin to deoxyhemoglobin [5]. The SpO_2_ to PaO_2_ relationship is linear at lower PaO_2_, but plateaus quickly at PaO_2_ > 80 mmHg, above which SpO_2_ will be 98–100% regardless of PaO_2_, consequently providing limited information regarding a patient’s PaO_2_ in the hyperoxic range. Conversely, SpO_2_ decreases rapidly as PaO_2_ falls below 80 mmHg and can fail to provide early detection of impending critical hypoxemia [4, 6, 7]. There are many clinical scenarios and patient factors that increase the risk for perioperative hypoxic events (SpO_2_ < 90%) including obesity [8, 9], difficult airway anatomy [10], rapid sequence induction/endotracheal intubation [11] and young age [12]. In these higher risk scenarios, detecting impending oxygen desaturation could allow interventions that might contribute to improved patient safety.

An infrared transmission pulse oximetry technology that utilizes ≥ 7 additional wavelengths of light transmission can determine a value called the Oxygen Reserve Index (ORI). ORI is reported on a unitless scale from 0 (minimal reserve) to 1 (high reserve) and correlates to PaO_2_ values in the mildly hyperoxic range between approximately 100 and 200 mmHg [4, 13, 14]. ORI is not a direct measure of PaO_2_ and there is wide variation in supra-normal PaO_2_ values after ORI values plateau [13, 15]. ORI has been shown to provide a clinically useful advanced warning of arterial hemoglobin desaturation in pediatric patients [16], during one lung ventilation [17], and during rapid sequence induction [18]. The ability of ORI to detect changes in arterial oxygenation within the mildly hyperoxic range could provide additional time to respond and consequently improve patient safety.

The prevalence of obese (body mass index (BMI) > 30 kg m^−2^) patients presenting for elective surgery is increasing [19]. It is predicted that by 2030 nearly 50% of the US adult population will be obese [20], while in some surgical populations obesity prevalence may reach 55% [21]. Obese patients have increased incidences of perioperative complications including atelectasis, difficult mask ventilation, difficult intubation and hypoxia [22–24]. Obese patients are prone to desaturate rapidly. This can be attributed to a combination of decreased oxygen reserve (decreased functional residual capacity), increased oxygen consumption, increased pulmonary resistance and airway obstruction [25].

This study was designed to determine if ORI can provide clinically significant increased warning time for impending desaturation compared to pulse oximetry in obese patients. To improve the clinical context and perspective we also evaluated the ORI and SpO_2_ warning times in patients with a normal BMI.

## Materials and methods

This prospective, observational study received Institutional Review Board approval, was registered at ClinicalTrials.gov (NCT03021551) and was conducted at University of California, Davis. All study procedures were performed in accordance with the ethical standards of the institutional and research committees and with the 1964 Helsinki declaration and its later amendments or comparable ethical standards. Written, informed consent was obtained from 40 patients with 30 < BMI < 40 kg m^−2^ (Obese) and 40 subjects with 19 < BMI < 25 kg m^−2^ (Normal BMI) scheduled for elective surgery with planned general anesthesia and endotracheal intubation. Exclusion criteria included patients with significant cardiopulmonary comorbidities, critically ill ICU patients, ASA > 4, and patients < 18 years of age. Anesthesia management was at the discretion of attending anesthesiologists responsible for patient care.

**Fig. 1:**
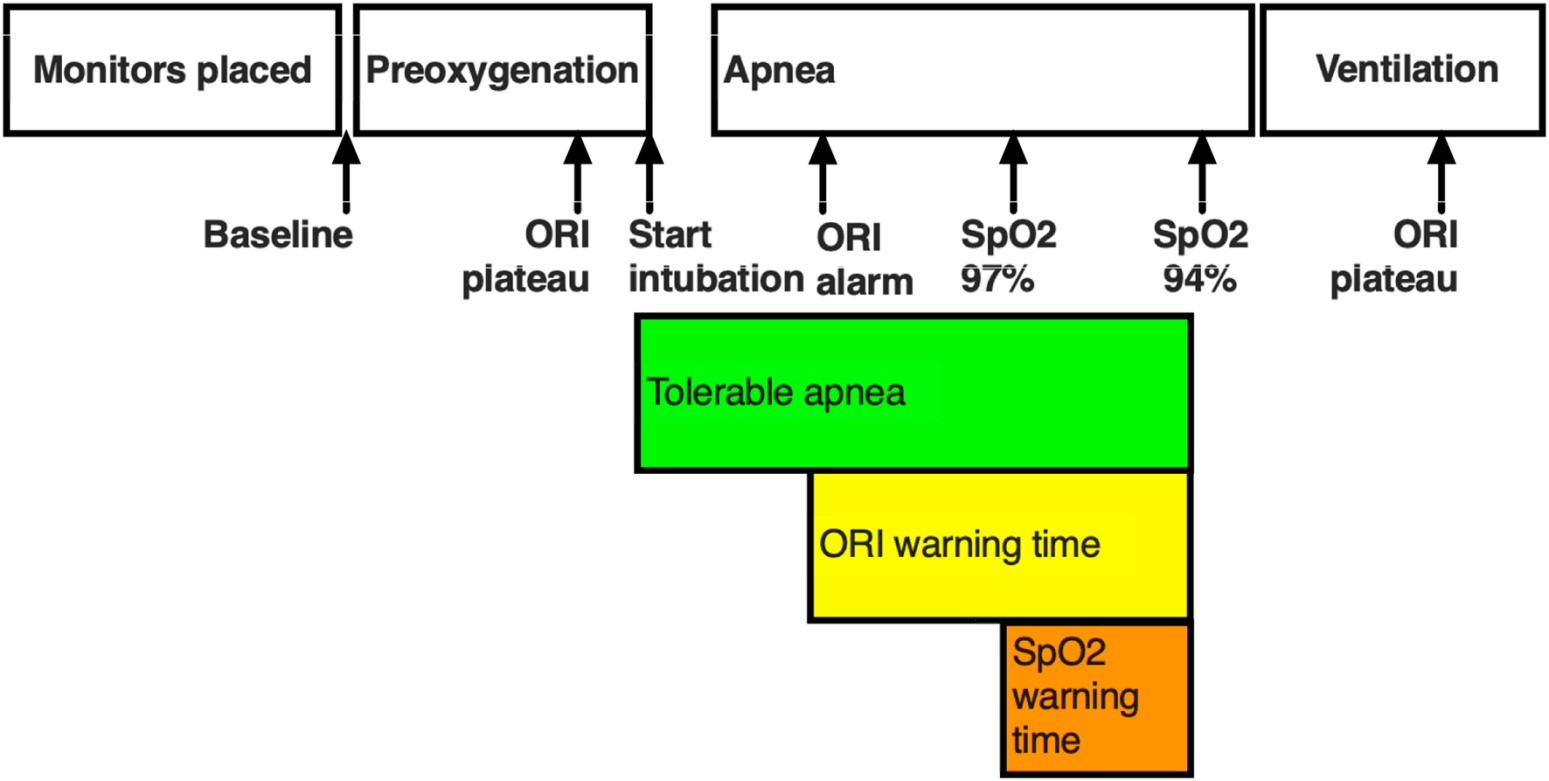
Oxygen Reserve Index (ORI) and pulse oximetry oxygen saturation (SpO_2_) data were compared at 5 timepoints: (1) baseline; (2) end of pre-oxygenation (ORI plateau); (3) start of intubation; (4) SpO_2_ = 94%; and (5) during ventilation with 100% FiO_2_ when ORI again reached a plateau

Patients were positioned supine on the operating room table and standard monitors were attached. In addition, an ORI oximetry sensor (Masimo Rainbow Disposable RD Lite) was placed on a finger, covered with an opaque shield to prevent exposure to ambient light and connected to a Masimo Root monitor with ORI measurement software. Data for analysis was subsequently downloaded directly from the Root monitor for analysis. After baseline vital signs were recorded, patient’s lungs were pre-oxygenated with spontaneous ventilation and 100% FiO_2_. Patients were encouraged to take repeated full vital capacity breaths until the ORI value plateaued. Thereafter, general anesthesia was induced using a combination of midazolam, fentanyl and propofol, ventilation was continued with positive pressure ventilation via face mask with 100% FiO_2_ and neuromuscular blocking drugs were administered. After establishing neuromuscular paralysis, the patients were intubated using a videolaryngoscope to verify appropriate positioning of the endotracheal tube. The breathing circuit was left detached from the endotracheal tube to avoid apneic oxygenation until the arterial saturation decreased to 94%. At that time, the breathing circuit was connected, and patients were ventilated with 100% FiO_2_, tidal volume targeted to 8 ml kg^−1^ ideal body weight and 5 cm H_2_O of positive end-expiratory pressure until ORI plateaued. Throughout this process, ORI and arterial saturation were continuously recorded using an automated data capture system (V3.2.2.0, copyright Masimo, Inc, 2015). ORI and SpO_2_ data were compared at five specific time points (Fig. 1): (1) baseline; (2) at the end of pre-oxygenation when the ORI reached a plateau; (3) at the start of intubation; (4) when SpO_2_ reached 94%; and (5) during ventilation with 100% FiO_2_ when ORI again reached a plateau.

**Fig. 2:**
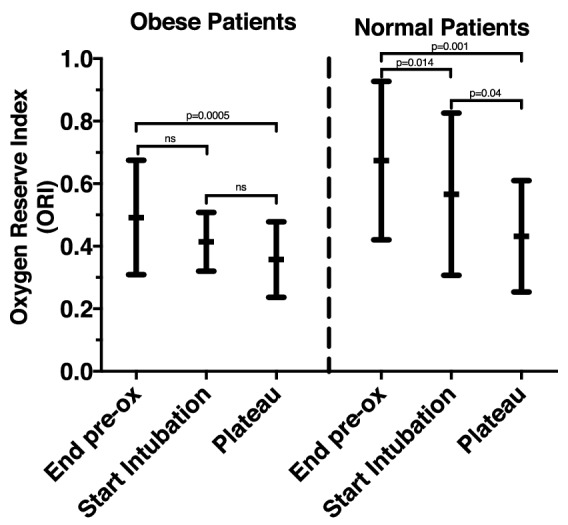
Comparison of Oxygen Reserve Index (ORI) values in Obese (30 < BMI < 40 kg m^−2^) and Normal BMI (19 < BMI < 25 kg m^−2^) patients at three measurement points: end of pre-oxygenation, start of intubation and plateau value following resumption of mechanical ventilation

The definitions used for comparisons of warning times represent a combination of clinical observations and pragmatic compromises. Prior publications have defined intraoperative desaturation events as SpO_2_ < 90% [2, 3, 12, 26]. However, World Health Organization training materials identify intraoperative SpO_2_ ≥ 95% as normal, and outline treatment steps for SpO_2_ ≤ 94% [27]. For this study, we took a conservative approach and defined the tolerable apnea as the time from the start of intubation until SpO_2_ reached 94% to provide some margin of safety. The ORI warning time was defined as the time between the start of the ORI alarm and SpO_2_ reaching 94%. The SpO_2_ warning time was defined as the time between 97% SpO_2_ and 94% SpO_2_ (Fig. 2).

### Statistical analysis

The statistical analysis plan was decided upon prior to subject enrollment. The primary outcome measure was the added warning time provided by ORI defined as the difference between ORI and SpO_2_ warning times. We defined a clinically significant difference in advance warning time as 30 s as the average time for successful endotracheal intubation by anesthesiologists using a videolaryngoscope to pass a single lumen endotracheal tube in studies included in a Cochrane review was just over 30 s [28]. To determine sample size, mean and standard deviation were estimated based on prior work that evaluated time for SpO_2_ to decrease from 98 to 90% [16], with alpha 0.05 and power of 0.8. Sample size to allow detection of a 30 s difference in warning time was 34 patients. We planned to enroll 40 patients each in the Obese and Normal BMI groups to allow for potential early withdrawal or data collection errors.

The distribution of the calculated times within each study group was tested using the D’Agostino & Pearson normality test. Data that were normally distributed were compared using t-test. The distributions of warning times and ORI values were not normal at each measurement event so statistical comparisons of the defined times and values were made using the Mann–Whitney test with the Hodges Lehman method used to determine differences between groups. For within group comparisons the Friedman test with Dunn’s multiple comparisons test was used. Categorical data were compared using Chi square or Fisher’s exact test. Analysis was performed using Prism (version 8.2.1, GraphPad Software, Inc, San Diego, CA, USA).

## Results

40 Obese and 40 Normal BMI subjects provided written, informed consent. In four Obese patients and 3 Normal BMI patients, the ORI sensor failed initial calibration. Clinical care time constraints precluded sensor adjustments directed to possibly correct this deficit and so no data were collected from these seven patients. In one Normal BMI patient, clinical care constraints precluded completion of the data collection as the surgeon was not willing to wait for development of the requisite level of desaturation prior to incision.

Patient characteristics are summarized in Table 1. Obese patients were older than Normal BMI (59 ± 14 vs 47 ± 17, p = 0.002) and had a smaller proportion of female patients (p = 0.011). The incidence of co-existing diseases was greater in Obese patients, but the distribution of American Society of Anesthesiologists Patient Status classifications was comparable between the two study groups (Chi-square p = 0.337). Small numbers in some patient groups limits the validity of this comparison.

**Table 1:** Patient characteristics

	Obese 30 < BMI < 40 kg m^−2^(n = 36)	Normal BMI 19 < BMI < 25 kg m^−2^ (n = 36)
Age years mean (range)	59 (25–82)	47 (21–75)
Sex # (%)		
Female	19 (53%)	30 (83%)
Male	17 (47%)	6 (17%)
Body mass index kg m^−2^ mean (range)	34 (30–39)	23 (19–25)
Co-existing disease (number)		
Cardiac	Hypertension (24)	Hypertension (2)
	Hyperlipidemia (15)	Hyperlipidemia (1)
	Coronary Artery Disease (3)	
	Congestive heart failure (1)	
Pulmonary	Asthma (8)	Asthma (5)
	Obstructive sleep apnea (8)	Obstructive sleep apnea (1)
	Active smoker (13)	Active smoker (6)
Renal	Chronic Kidney Disease (3)	
Gastrointestinal	Gastroesophageal reflux (14)	Gastroesophageal reflux (6)
Endocrine	Hypothyroidism (3)	Hypothyroidism (8)
	Diabetes (type I) (1)	
	Diabetes (type II) (6)	Diabetes (type II) (2)
ASA physical status #1/2/3/4	2/15/17/2	5/19/11/1
Types of operation		
Cardiothoracic	2	2
Head and neck	10	6
General	14	12
Gynecologic	5	7
Neurosurgery	0	1
Oncologic	2	1
Orthopedic	0	1
Plastic	0	3
Urologic	2	0
Vascular	0	2

ORI values varied widely both within and between study groups. Figure 2 summarizes ORI changes within each study group. In Obese patients, ORI values at the end of pre-oxygenation did not differ from those at the start of intubation (0.49 ± 0.18 vs 0.41 ± 0.09 but did decrease slightly when ventilation resumed (0.36 ± 0.12). In contrast, ORI values for Normal BMI patients decreased between the end of pre-oxygenation and the start of intubation (0.67 ± 0.25 vs 0.57 ± 0.26) and decreased further when ventilation was resumed (0.43 ± 0.18). Between study group comparisons are summarized in Table 2. ORI values for Obese patients were lower than those for the Normal patients both at the end of pre-oxygenation and the start of intubation.

**Table 2:** Oxygen Reserve Index Values in Obese (30 < BMI < 40 kg m^−2^) and Normal BMI (19 < BMI < 25 kg m^−2^) patients

	End pre-oxygenation		Start intubation		Post-ventilation plateau	
	Obese	Normal BMI	Obese	Normal BMI	Obese	Normal BMI
Mean	0.49	0.67	0.41	0.57	0.36	0.43
SD	0.18	0.25	0.09	0.26	0.12	0.18
95% CI	0.43 to 0.55	0.59 to 0.76	0.38 to 0.45	0.48 to 0.65	0.32 to 0.40	0.37 to 0.49
p-value	0.003		0.018		0.051	

**Fig. 3:**
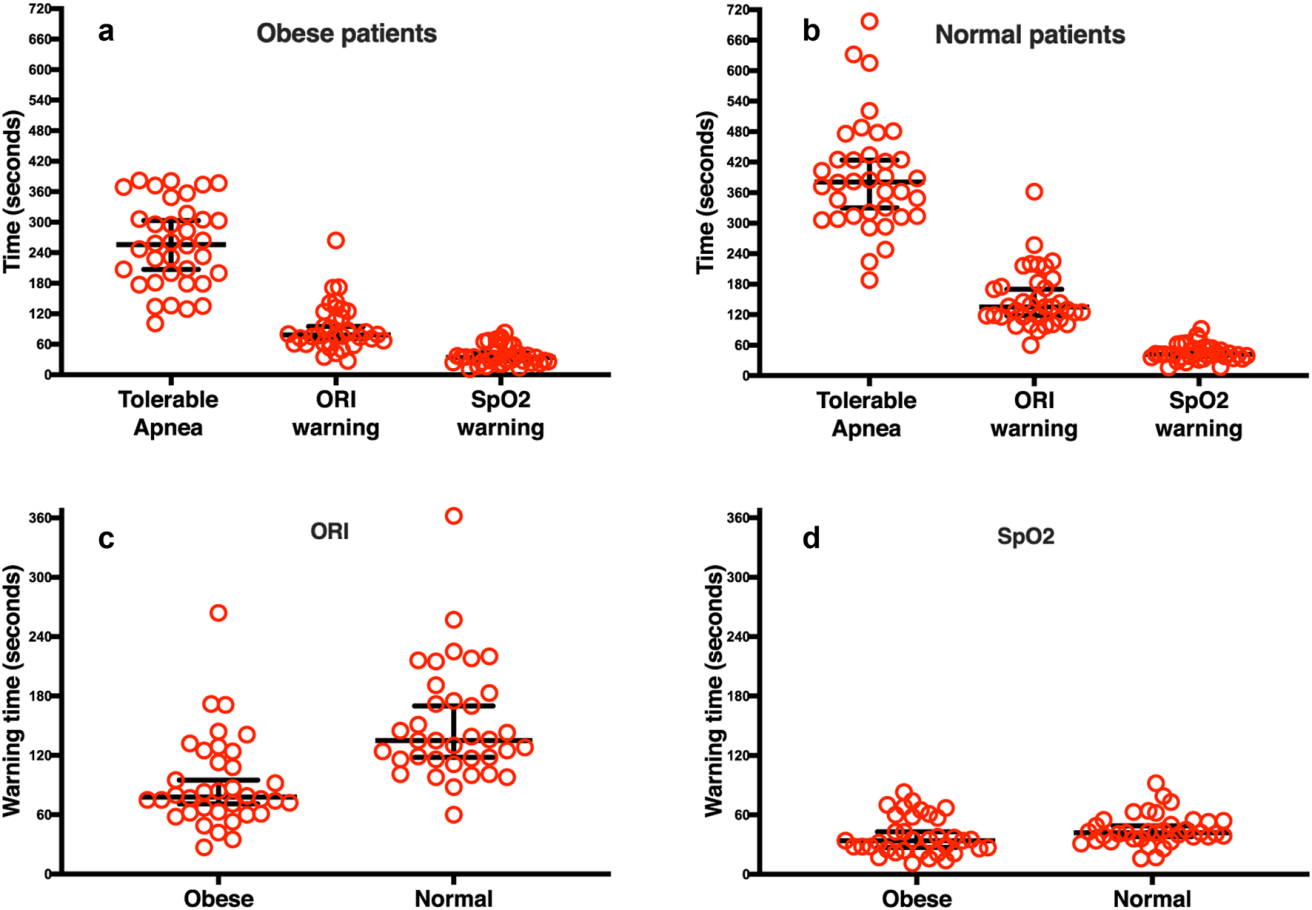
Comparison of defined study times. **a** Comparison of tolerable apnea to warning times provided by Oxygen Reserve Index (ORI) and pulse oximetry oxygen saturation (SpO_2_) in Obese patients (body mass index (BMI); 30 < BMI < 40 kg m^−2^); **b** Comparison of tolerable apnea to warning times provided by ORI and SpO_2_ in Normal BMI patients (19 < BMI < 25 kg m^−2^). ORI warning time was longer than SpO_2_ warning time in all patients; **c** Comparison of warning times in Obese patients to those in Normal BMI patients provided by ORI; and **d** comparison of warning times in Obese patients to those in Normal BMI patients provided by SpO_2_

The time results for each of the defined periods are summarized in Table 3. In all patients the ORI alarm began before SpO_2_ reached 97% so the ORI warning time was always longer than SpO_2_ warning time. As illustrated in Fig. 3, ORI provided significant added warning time compared to SpO_2_ in Obese (added warning time = 46.5 s; 95% CI 36.0 to 59.0 s; p < 0.0001) and Normal BMI patients (added warning time = 87.0 s; 95% CI 77.0 to 109.0 s; p < 0.0001). The added warning time provided by ORI was shorter in Obese than Normal BMI patients (difference 54.0 s; 95% CI 38.0 to 74.0 s; p < 0.0001). While not clinically significant, the SpO_2_ warning time was shorter in Obese than in Normal BMI patients (difference 8.0 s; 95% CI 1.0 to 15.0 s; p = 0.036). The tolerable apnea time was approximately 2 min shorter in Obese than in Normal BMI patients (difference 126.0 s; 95% CI 83.0 to 173.0 s; p < 0.0001).

**Table 3:** Comparison of defined study times in Obese (30 < BMI < 40 kg m^−2^) and Normal BMI (19 < BMI < 25 kg m^−2^) patients**.**

Time in seconds	Obese n = 36	Normal BMI n = 36	Difference 95% CI	p
Tolerable apnea Median 25–75th percentile	256.0 185.8 to 256.0	381.0 314.0 to 431.8	126.0 83.0 to 173.0	< 0.0001
ORI warning time Median 25–75th percentile	78.0 62.3 to 121.3	135.0 116.0 to 181.0	54.0 38.0 to 74.0	< 0.0001
SpO_2_ warning time Median 25–75th percentile	34.0 24.3 to 57.8	42.0 36.0 to 53.8	8.0 1.0 to 15.0	0.036
Added warning time Median 25–75th percentile	46.5 33.3 to 63.8	87.0 71.3 to 129.3	44.0 28.0 to 61.0	< 0.0001

## Discussion

We found ORI provides a clinically significant added warning time for impending desaturation in both Obese and Normal BMI patients. Pulse oximetry is an invaluable tool for intraoperative arterial oxygenation monitoring [3, 29] because of the consistent and well-described relationship with PaO_2_. Accurate monitoring of intraoperative oxygenation status is imperative, especially in high-risk populations such as obese patients who are prone to rapid desaturation. However, because of the sigmoidal nature of this relationship, SpO_2_ may not warn of impending critical arterial deoxygenation until PaO_2_ is less than about 80 mmHg. Although not tested in this study, the added warning time of more than 46 s prior to any appreciable decrease in SpO_2_ in obese patients could result in improved patient outcomes as it should allow earlier calls for help and more time for modifications of airway management plans. Of interest, when using the ORI plateau as an indicator guide of preoxygenation, Normal BMI patients tolerated longer periods of apnea than obese patients.

Prior studies found ORI to provide a clinically significant median warning time of 31.5 s in pediatric [16] patients and 201 s in adults during one lung ventilation [17] prior to any substantial changes in SpO_2_. Our findings support the potential utility of ORI monitoring in obese patients as an adjunct to standard monitoring in the operating room environment. The potential clinical impact of this monitor might be extrapolated from the incidence of intraoperative hypoxemia. A study of 95,407 electronic patient records from two large academic hospitals found a 6.8% incidence of hypoxic events for two minutes or longer during the intraoperative period [2], consistent with other studies [2, 12, 26]. Peri-operative hypoxia has been shown to have deleterious effects on end-organ perfusion, which can lead to worse patient outcomes. Although the severity and threshold duration of hypoxic events necessary to worsen outcome is unknown, hypoxic injury has been implicated as contributing to higher postoperative complications in thoracic surgery [30], cognitive dysfunction [31, 32], acute renal failure [33], myocardial ischemia [34] and pulmonary hypertension [35].

In healthy young patients critical SpO_2_ desaturation is more likely to provide adequate time for necessary interventions, while higher risk and obese patients have a propensity to desaturate more rapidly, leaving less time for addressing evolving hypoxemia [25]. The prevalence of obesity (BMI > 30 kg m^−2^) is increasing, and as demonstrated in this study, obese patients often have additional comorbid conditions and present for a variety of surgical procedures [19]. Furthermore, obese patients have a higher incidence of difficult mask ventilation and endotracheal intubation [23] making common interventions more difficult and less likely to be successful. In this context, any additional advanced warnings have potential benefits. Additional safety can be gained by optimal pre-oxygenation which is imperative to maximize the apneic time prior to critical desaturation during endotracheal intubation. These data suggest that ORI may provide useful guidance and benefit in this arena as well.

There are some limitations to generalization of our findings. We encountered a relatively large number of sensor failures in this study (7/80). We do not believe this limits the utility of the monitor. The majority of these failures occurred early in the course of this study before we became aware of the importance of proper alignment of the emitters and receivers when placing this sensor. To minimize the impact of this trial on the clinical care flow we did not have the time available to adjust sensor placement in these cases. There were also some differences in the demographics of the two study groups. Patients in the Obese group were older, included a greater proportion of males, had an increased incidence and diversity of co-existing diseases and there were differences in the distribution of the planned surgical procedures. All of these factors had the potential to influence the efficacy of pre-oxygenation and the rate of desaturation. We believe that in the comparisons of these two groups, the BMI was the predominant determinant of the rate of hemoglobin desaturation and that the relative impacts on ORI and SpO_2_ were similar. These other differences might have contributed to the overall variability of the tolerable apnea time. We did not standardize the preoxygenation period. Measurements were initiated when the ORI value plateaued. In the Obese patients, this required and average of 3.3 ± 1.4 min and in patients with a Normal BMI this required a comparable 3.1 ± 1.2 min. End-tidal oxygen concentrations at the end of preoxygenation were not recorded for this study but were generally > 90%. The previously demonstrated wide ranges of supra-normal PaO_2_ values that are associated with maximal ORI values [13, 15] suggests the potential for wide variations in total oxygen reserves and consequent increased variability in the total time of tolerable apnea. In addition, following induction of general anesthesia, manual ventilation was continued until laryngoscopy for a total time of ventilation with 100% oxygen of 6.6 ± 1.9 min in the Obese patient group and 7.2 ± 2.0 min in the patients with a Normal BMI. No patient required supplemental CPAP, BiPAP or PEEP to maintain adequate oxygenation. Although these variations also have the potential to impact the total tolerable apnea period, they are not likely to change the relative differences between ORI and SpO_2_ warnings as each patient serves as their own control. In addition, to allow objective comparisons, the ORI alarm was used to initiate the start of the ORI warning time. This alarm is triggered by a proprietary algorithm based upon both the absolute ORI value and the rate of decline. Other studies of the clinical utility of the ORI have used either percentage or absolute value changes as the start of the advanced warning. Although this could possibly increase the absolute advanced warning time provided by the ORI and perhaps decrease the number of outlier measurements, we believe the comparative relationship to the SpO_2_ warning would be similar. Furthermore, clinicians can’t be blinded to the Obese or Normal BMI patient categories compared in this trial. This may have influenced induction choices, which were not controlled. This is difficult to detect or quantify but seems unlikely to have impacted the difference in ORI and SpO_2_ warning times in Obese patients as calculated by this protocol. Lastly, our study did not evaluate interventions carried out within any added ORI warning time, so further research is necessary to determine if this ORI warning time impacts patient care outcomes.

Careful monitoring of oxygenation during critical care periods such as anesthesia should help to avoid complications associated with arterial hypoxemia. Our findings suggest ORI could serve as a useful adjunct to SpO_2_ in patients in whom 30 < BMI < 40 kg m^−2^, and aid patient safety since the added warning time may allow for improved airway management and earlier calls for assistance from other experienced providers. Further analysis of the correlations of ORI and PaO_2_, the use of ORI as a guide to pre-oxygenation and its utilization in the morbidly obese (BMI > 40 kg m^−2^) patients need further study.
